# Improving Radiotherapy Plan Quality for Nasopharyngeal Carcinoma With Enhanced UNet Dose Prediction

**DOI:** 10.1002/cam4.70688

**Published:** 2025-02-15

**Authors:** Junming Jian, Xingxing Yuan, Longfei Xu, Changfei Gong, Xiaochang Gong, Yun Zhang

**Affiliations:** ^1^ Department of Radiation Oncology Jiangxi Cancer Hospital & Institute (The Second Affiliated Hospital of Nanchang Medical College) Nanchang Jiangxi People's Republic of China; ^2^ Jiangxi Clinical Research Center for Cancer Nanchang Jiangxi People's Republic of China; ^3^ Jiangxi Key Laboratory of oncology (2024SSY06041) Nanchang Jiangxi People's Republic of China

**Keywords:** deep learning, dose prediction, nasopharyngeal carcinoma, volumetric modulated arc therapy

## Abstract

**Background:**

Individualized dose prediction is critical for optimizing radiation treatment planning. This study introduces *DESIRE*, an enhanced UNet‐based dose prediction model with progrEssive feature fuSion and dIfficult Region lEarning, tailored for nasopharyngeal carcinoma (NPC) patients receiving volumetric modulated arc therapy. We aimed to assess the impact of integrating *DESIRE* into the treatment planning process to improve plan quality.

**Methods:**

This retrospective study included 131 NPC patients diagnosed at Jiangxi Cancer Hospital between 2017 and 2020. Twenty patients were randomly allocated to a testing cohort, while the remaining 111 comprised a training cohort. Target delineation included three planning target volumes (PTVs): PTV70, PTV60, and PTV55, along with several organs at risk (OARs). The *DESIRE* model predicted dose distributions, and discrepancies between *DESIRE*'s predictions and the ground truth (GT) were quantified using dosimetric metrics and gamma pass rates. Two junior physicians used *DESIRE*'s predictions for treatment planning, and their plans were compared to the GT.

**Results:**

Most of *DESIRE*'s predicted dosimetric metrics closely aligned with GT (mean difference < 1 Gy), with no significant differences (*p* > 0.05) in *D*_*mean*_ and *D*_*1*_ values across OARs. While significant differences were observed in PTV metrics, the mean differences in *D*_*98*_, *D*_*95*_, *D*_*50*_, and *D*_*mean*_ between *DESIRE* and GT did not exceed 1 Gy. Assisted by *DESIRE*, the junior physicians' plans were comparable to the GT in nearly all OARs, with no significant differences in dosimetric metrics. The conformity index (CI) and homogeneity index (HI) for PTV70 surpassed the GT (0.847 ± 0.036 vs. 0.827 ± 0.037 for CI, and 0.057 ± 0.009 vs. 0.052 ± 0.008 for HI). The average three‐dimensional gamma passing rates were 0.85 for PTV70 and 0.87 for the 35‐Gy isodose line.

**Conclusions:**

The *DESIRE* model shows promise for patient‐specific dose prediction, enhancing junior physicians' treatment planning capabilities and improving plan quality.

## 1 Background

Radiation therapy (RT) is a crucial method for cancer treatment, working to control the spread of cancer using high‐energy radiation to kill malignant cells. In recent years, there have been remarkable improvements in the treatment planning quality due to the introduction of intensity‐modulated radiation therapy (IMRT) and volumetric‐modulated arc therapy (VMAT) [1, 2]. However, this progress comes with the trade‐off of increased complexity, especially when planning therapy in intricate areas, and it often requires multiple attempts and invests a substantial amount of time to enhance plan quality.

Researchers have been actively working to address these challenges. Knowledge‐based planning (KBP) methods [3, 4], widely applied in recent years, primarily rely on historical treatment planning data for each patient as a reference to create dose distribution plans for new clinical treatments. The dose–volume histogram (DVH) [5] is a clinical evaluation metric that is commonly used in KBP methods, which can help automate radiotherapy planning. However, KBP methods still have several critical limitations. First, they rely heavily on handcrafted features corresponding to the dose distribution [6]. The extraction of features is not only timeconsuming but also lacks accuracy. Second, the features extracted by DVH lack spatial details about dose distributions [7], due to the compression and asymmetric mapping of three‐dimensional (3D) dose information. Moreover, these methods fail to consider anatomical structures and location information around tumors, thus neglecting the influence of surrounding organs.

Most recently, convolutional neural networks (CNNs) have made remarkable breakthroughs in medical image analysis [8, 9], leading to their application in radiotherapy dose prediction, with 3D UNet and its variants being the predominant models. Nguyen et al. [10] and Alexander et al. [11] proposed hierarchically densely connected UNet and attention‐aware 3D UNet, respectively, for IMRT 3D dose distribution prediction in head and neck cancer. Li et al. [12] proposed a multitask attention adversarial network to automatically complete dose planning in cervical cancer, in which UNet served as the main architecture for the dose decoder and auxiliary segmentation. Similar studies can also be found in breast cancer [7], colorectal cancer [13], and prostate cancer [14].

Despite these successes, there is room for improvement in prediction accuracy. First, progressive feature fusion contributes to enhancing the depth and effectiveness of features, thus improving the modeling capability. Second, difficult regions should be considered to prevent models from taking shortcuts by optimizing only for easy areas, improving clinical relevance. Furthermore, with specifically designed training constraints, models can be explicitly guided to focus on high‐dose regions, leading to increased dose prediction accuracy. Finally, most current research efforts mainly aim to enhance dose prediction accuracy. Nonetheless, a considerable lack of investigations exists on how dose prediction can influence the quality of planning, a crucial aspect of clinical care.

To this end, we propose herein *DESIRE*, a *Dose prediction model with progrEssive feature fuSion and dIfficult Region lEarning* in this study, illustrating its use in a study of patients with nasopharyngeal carcinoma (NPC) after VMAT. First, a progressive UNet was designed for feature extraction and effective information fusion, incorporating a residual dense (RD) block to enhance learning capabilities further. Moreover, to explicitly enforce the constraints closely related to the dose prediction task, we proposed a novel loss function that can guide the model to focus on difficult regions and high‐dose areas during training. We compared our proposed *DESIRE* model with several state‐of‐the‐art methods using an indoor validation dataset from patients with NPC to validate its feasibility in dose prediction. Meanwhile, two junior physicians were invited to reference the dose prediction results of the *DESIRE* for treatment planning. The quality of their plans was compared with that of senior physicians to assess the potential clinical value of the model.

## 2 Methods

### 2.1 Patient Enrollment

This retrospective study included 131 patients diagnosed with NPC between 2021 and 2023 at Jiangxi Cancer Hospital. The median age of the patients was 55 years (range 23–87 years), and they presented with stages T2–T4 malignancies. This study was approved by the Medical Ethics Committee of our hospital, and informed consent was waived due to its retrospective nature. The patients were positioned in a supine posture and immobilized with a thermoplastic mask combined with a custom mold on the Solo Align Full Body System (Klarity, China). All CT images were acquired using the Siemens SOMATOM Definition AS CT machine (Siemes, Erlangen, Germany). CT images had a slice thickness of 3 mm and a size of 512 × 512 pixels. There were four target areas in this study: (1) GTVnx, including the visible primary tumor site and its extent as observed on imaging and clinical examination; (2) GTVnd, including the gross tumor volume with neck lymph node metastases; (3) CTV1, including GTVnx plus a 5–10‐mm margin, as well as the corresponding nasopharyngeal mucosa and the submucosa within 5 mm; and (4) CTV2, encompassing CTV1 and, based on tumor invasion and extent, the posterior nasal cavity, posterior maxillary sinus, pterygopalatine fossa, some posterior ethmoid sinuses, parapharyngeal space, skull base, or some cervical vertebrae. An additional 3‐mm margin was added around each of the four target areas to create PTVnx, PTVnd, PTV1, and PTV2. The prescribed dose for PTVnx was 70 Gy, for PTVnd was 70 Gy, and for PTV1 and PTV2, it was 60 Gy and 55 Gy, respectively. For simplification in the subsequent analysis, PTVnx and PTVnd were consolidated into a single entity termed PTV70; PTV1 is referred to as PTV60, and PTV2 as PTV55. The OARs included the brain stem, eyes (L, R), lens (L, R), middle ear (L, R), optic nerve (L, R), parotid (L, R), temporal lobes (L, R), temporomandibular joint (L, R), larynx, optic chiasma, oral cavity, and spinal cord.

Because the original plans were created with varying levels of planning, their quality varied. However, the model training results depended on the original plans to guide subsequent lower‐level plans for generating future plans using referenced predicted doses. Thereby, each patient's VMAT plan was re‐generated by a planner with more than 10 years of planning experience using a 3D treatment planning system. All VMAT plans were generated using Eclipse software (version 13.5) with two arcs using a 6‐MV photon beam from a TrueBeam linear accelerator (Varian Medical Systems). The optimization goal was to ensure that at least 95% of the planning target volumes (PTVs) would receive the prescribed dose and no more than 5% of the PTV70 would receive 107% of the prescribed dose while minimizing the dose to OARs. The final doses were calculated using the Acuros XB algorithm with a 2.5‐mm grid resolution. An oncologist assessed the quality of each treatment plan regarding its clinical acceptability.

### 2.2 Data Preparation

Each patient's data included CT images, annotations of the target areas and OARs, and the radiotherapy plan dose. All CT images and dose distributions were rescaled to the size of 96 × 128 × 128. Subsequently, CT values were truncated to the range of (−1024, 2048) and then normalized to (0, 1). As for dose distributions, they were divided by 70 grays.

### 2.3 Model and Training

#### 2.3.1 Overall Model Architecture

**FIGURE 1:**
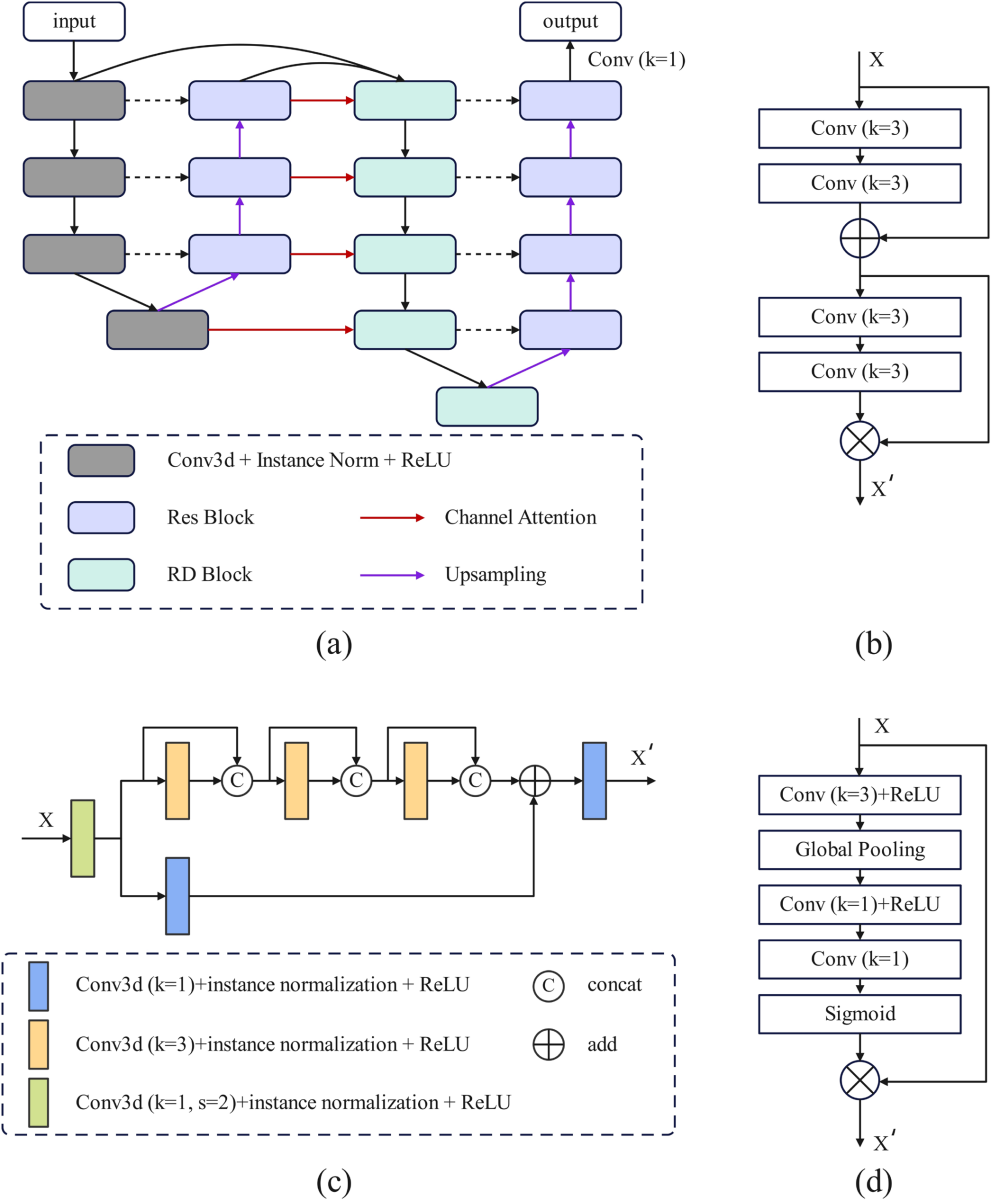
Model architecture of proposed *DESIRE* model, (a) overall, (b) residual block, (c) residual dense block, and (d) channel attention.

As shown in Figure 1, *DESIRE* consists of two progressive UNet architectures (Figure 1a). It starts by employing a 4‐layer UNet structure for initial feature extraction. Subsequently, a channel attention module (Figure 1d) transforms the extracted features of the four upsampling layers into attention weights, which are then applied to the second UNet. The second UNet is a five‐layer structure, with each layer having twice the number of channels as the corresponding layer in the first UNet. Its input consists of the features from the previous layer and the corresponding feature generated from the first UNet. In this way, more effective feature extraction and fusion can be achieved in terms of both network depth and width.

Both encoders of the two UNets replace the max‐pooling layers with convolution layers with a stride of two for down‐sampling operations. The first encoder utilizes a standard 3D convolution module, whereas the second encoder employs an RD block (Figure 1c) to enhance feature extraction. For the decoder, trilinear interpolation is used for feature upsampling, and feature fusion is implemented by residual modules (Figure 1b). Finally, a 1 × 1 × 1 convolution is used to generate the final prediction.

#### 2.3.2 Residual Dense Block

The residual connections of ResNet and the dense connections from DenseNet are effectively integrated into a new RD block, as illustrated in Figure 1c. The RD block begins by down‐sampling through a 3D convolution with a stride of two. Afterward, two separate processing branches are used for different feature transformations. The upper branch consists of three convolution modules, each concatenating the original feature and transformed features generated by current convolution operations. For the other branch, a 1 × 1 × 1 convolution is used to obtain local features. The results from both branches are then fused by element‐wise summation, followed by another 1 × 1 × 1 convolution. In this way, the global features from the upper branch and the local features from the lower branch are integrated. All the convolution operations are followed by instance normalization and a ReLU activation function.

#### 2.3.3 Difficult Region Learning

**FIGURE 2:**
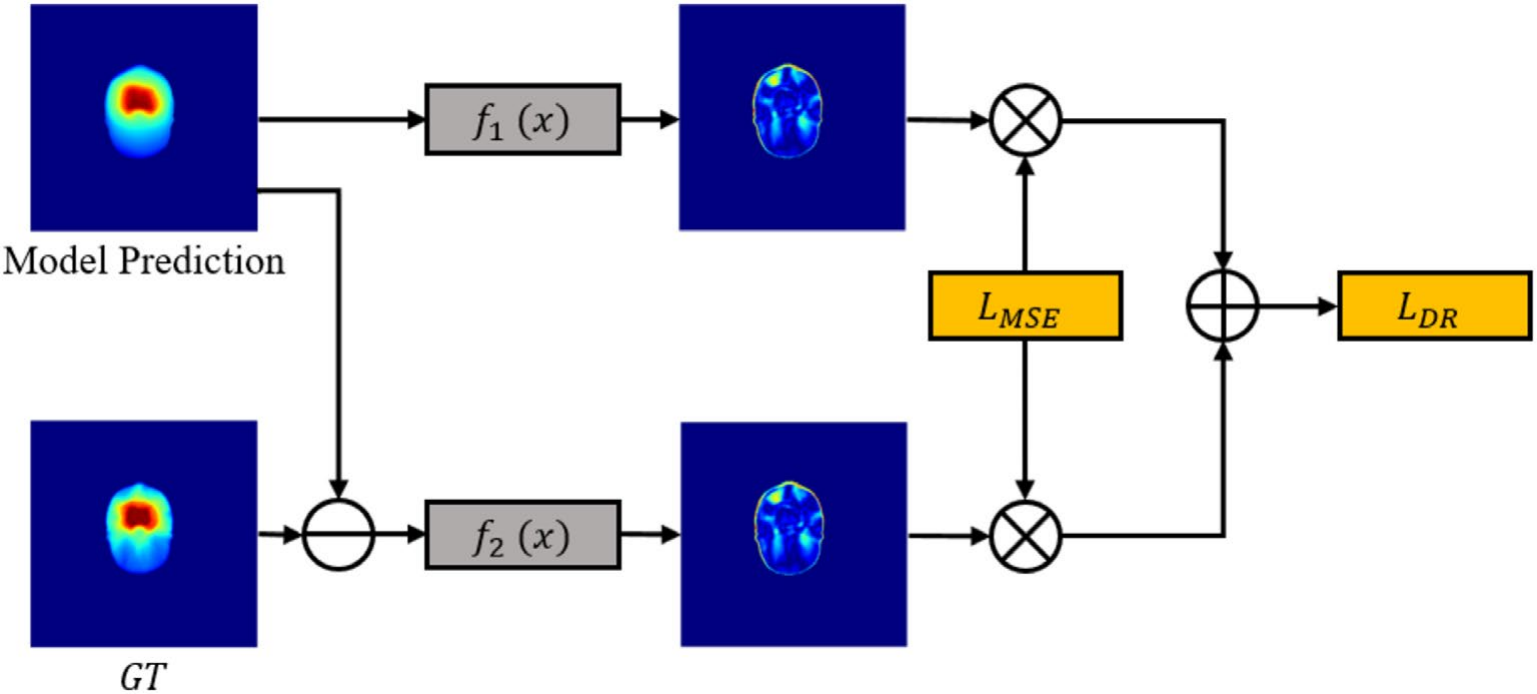
Difficult region learning loss.

Dose prediction is a pixel‐level regression task. With currently widely used loss functions, such as the mean average error loss, CNNs are prone to pay more attention to the regions that are easy to learn while ignoring more difficult, but important, areas. We introduced difficult region learning loss to explicitly guide the model to be more attentive to difficult regions, as illustrated in Figure 2.

First, we used the mean squared error to calculate the difference, denoted as $L_{\mathrm{MSE}}$, between the model prediction and GT. Next, we substitute the model's standard output with four dropout outputs, each with distinct probability distributions. The objective is to empower the model to acquire four outputs characterized by enhanced diversity and randomness in a single forward propagation iteration. The four dropout outputs are separately processed through two branches. In the first branch, the four dropout outputs are processed with the function $f_{1}$ to calculate the variance and normalization, resulting in the first weight map. The formula for $f_{1}$ is shown in Equation (1). In the second branch, we calculate the mean of the four dropout outputs and subtract it from the GT. Then, with the function $f_{2}$, as shown in Equation (2), the second weight map is obtained. Afterward, the ${\text{Loss}}_{\mathrm{MSE}}$ is used to calculate the difference between the generated two weight maps, which is then used to obtain the final difficult region learning loss $L_{\mathrm{DR}}$, as shown in Equation (3). Here, m is the number of samples, $d_{1}$, $d_{2}$, $d_{3}$, and $d_{4}$ are the results of the four dropout predictions, $\mathrm{Var}\left(D\right)$ is their variance, $\text{Mean}\left(D\right)$ is the average, and α and β are the hyper‐parameters for proper adjustment of the normalization mapping and pay more attention on difficult regions. In our experiments, we experimentally set α and β as 0.5 and 0.05, respectively.

(1) $$f_{1}=f\left(d_{1},d_{2},d_{3},d_{4}\right)=\frac{\mathrm{Var}\left(D\right)}{\alpha +\mathrm{Var}\left(D\right)}$$

(2) $$\begin{array}{c} f_{2}=f\left(d_{1},d_{2},d_{3},d_{4}\right)=\frac{\left|\text{Mean}\left(D\right)-\mathrm{GT}\right|}{\beta +\left|\text{Mean}\left(D\right)-\mathrm{GT}\right|} \end{array}$$

(3) $$\begin{array}{c} L_{\mathrm{DR}}=\frac{1}{m}\sum_{n=1}^{m}\left(f_{1}+f_{2}\right)∙{\left|y_{n}-\hat{y_{n}}\right|}^{2} \end{array}$$

#### 2.3.4 Auxiliary Segmentation Loss

**FIGURE 3:**
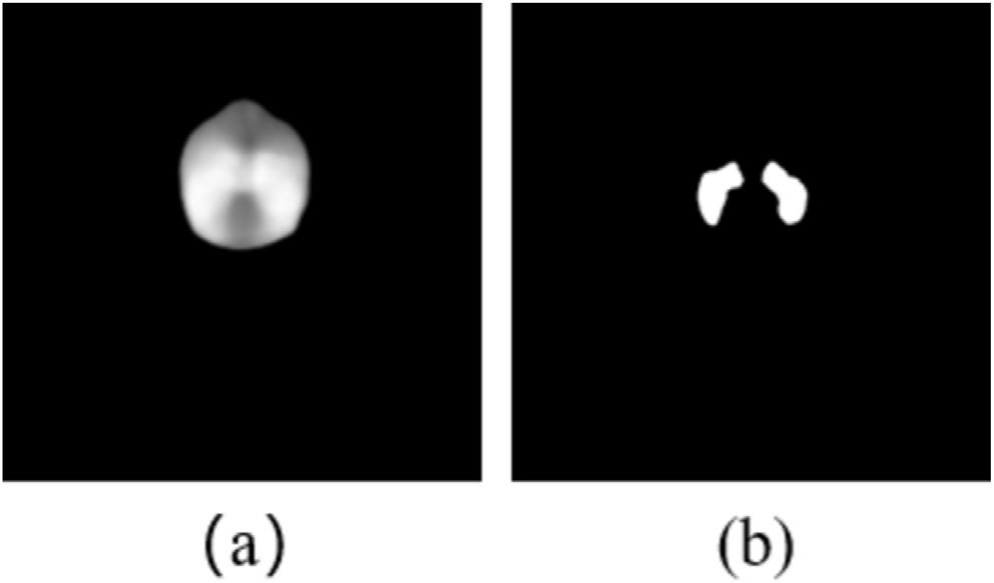
Dose distribution map (a) and corresponding high‐dose regions (b).

For the dose distribution map, there are regions where the values exceed the prescribed dose, which require more attention weight. Therefore, a semantic segmentation loss function is employed to enforce the model to focus on the high‐dose regions explicitly. Figure 3a represents the dose distribution map, with a prescription dose of 70 Gy for PTVnx. We labeled the regions where the dose is greater than or equal to 70 Gy as the high‐dose regions (Figure 3b). The combination of the binary cross‐entropy (BCE) and Dice loss functions is used to compute the difference between the dose prediction results and the GT within those high‐dose regions:

(4) $$\begin{array}{c} L_{\mathrm{seg}}=\frac{1}{k}\sum_{n=1}^{k}\left(0.5\times {\text{BCELoss}}_{n}+0.5\times {\text{DICELoss}}_{n}\right) \end{array}$$

Here, k denotes the number of high‐dose regions.

#### 2.3.5 Final Loss

The final loss $L_{\text{final}}$ is the combination of mean absolute error (MAE) loss $L_{\mathrm{MAE}}$, difficult region learning loss $L_{\mathrm{DR}}$, and the auxiliary segmentation loss $L_{\mathrm{seg}}$:

(5) $$\begin{array}{c} L_{\text{final}}=L_{\mathrm{MAE}}+L_{\mathrm{DR}}+\gamma \times L_{\mathrm{seg}} \end{array}$$

Here, γ adjusts the weight of the auxiliary segmentation loss, and we experimentally set it as 0.1 to prevent an excessively high auxiliary segmentation loss weight, which could decrease the final dose prediction performance.

### 2.4 Experimental Settings

All the experiments were implemented using PyTorch (v1.10) and conducted with NVIDIA TITAN RTX (24 GB). We trained our model using the Adam optimizer with a batch size of one for 150 epochs. The model parameters were initialized using the Kaiming method [15]. The model's initial learning rate was set to 0.0003, and during training, it gradually decreased using a cosine annealing with warm restarts strategy [16].

### 2.5 Model Evaluation

To assess our model's performance, we used several dosimetric metrics, encompassing *D*_2_, *D*_50_, *D*_95_, *D*_98_, and *D*_*mean*_. Here, *D*_*x*_ denotes the absorbed dose covering x% of the PTV, whereas *D*_*mean*_ represents the average dose delivered to the PTV. Specifically, all the metrics were utilized to assess PTV70, PTV60, and PTV55, whereas *D*_*1*_ and *D*_*mean*_ were employed to evaluate OARs.

To directly quantify the dose disparity between the GT and the prediction results of the models, we calculated the MAE.

(6) $$\begin{array}{c} \mathrm{MAE}\left(\hat{y},y\right)=\frac{1}{n}\sum_{i=1}^{n}\left|{\hat{y}}_{i}-y_{i}\right| \end{array}$$

where *n* represents the total number of voxels ${\hat{y}}_{i}$ and $y_{i}$ represent the GT and predicted values. Meanwhile, the results were compared with 2D‐UNet [17], HD‐UNet [10], and 3D‐UNet [18].

In addition, we employed global three‐dimensional gamma analysis to assess the accuracy of dose distribution predictions for each OAR. Consistency was evaluated at a dose threshold value set at 10% of the prescription dose and a tolerance level of 3%/3 mm [19].

To further explore the clinical relevance of the model for treatment planning, we engaged two junior physicians, one with fewer than 3 years of experience and the other with fewer than 5 years of experience, to review the dose prediction results of *DESIRE*. For the planning design phase, we meticulously selected 20 cases used for model testing. The initial step involved the assessment of DVH curves for dose predictions and specific 3D dose distributions by two less experienced planners. Subsequently, these planners adjusted the dose‐limiting regions derived from the standard template, informed by the aforementioned data. They then individually tailored the optimization objective function for identified cold spot regions and set personalized target optimization parameters and optimization weights for normal tissues, guided by DVH curves. Notably, in areas where the target region overlapped with healthy tissue, optimization objectives were delineated based on the anticipated dose distribution, catering to each specific area. Following the establishment of individual optimization parameters, the planning optimization commenced, with periodic revisions of the optimization target based on the attained results. This iterative process aimed to ensure that the target area coverage aligned with clinical requirements while minimizing dose exposure to healthy tissue.

The quality of the plans was then compared with those of senior physicians (i.e., GT) to evaluate the model's clinical utility. In addition to the aforementioned metrics, the conformity index (CI) and the heterogeneity index (HI) were also included in this comparison. CI is defined as:

(7) $$\begin{array}{c} \mathrm{CI}=\frac{V_{\mathrm{ref}}}{V_{\mathrm{ptv}}}\times \frac{V_{\mathrm{ref}}}{V_{\text{pres}}}\; \end{array}$$

where $V_{\mathrm{ptv}}$ and $V_{\text{pres}}$ are the volume of PTV and the prescription dose region, respectively, and $V_{\mathrm{ref}}$ is the irradiated PTV volume of the prescription dose. A higher similarity between the GT and prediction map indicates better model performance. HI is defined as follows:

(8) $$\begin{array}{c} \mathrm{HI}=\frac{D_{2}-D_{98}}{D_{50}} \end{array}$$

In contrast to CI, a smaller HI value indicates better prediction results.

### 2.6 Statistical Analysis

All statistical analyses were performed using Python (version 3.10.13; https://www.python.org/). The differences between the *DESIRE* and the GT, between models, and between planners were all assessed using the Mann–Whitney *U* test. *p*‐values < 0.05 were considered statistically significant.

## 3 Results

### 3.1 Dosimetric Metrics Comparison

Table 1 demonstrates the dosimetric metric differences for PTVs between the dose distribution predicted by *DESIRE* and the corresponding metrics of the actual dose distribution, expressed as means ± standard deviations. The *D*_*95*_ values for *DESIRE* concerning PTV70, PTV60, and PTV55 were 70.03 Gy, 62.13 Gy, and 55.67 Gy, respectively, which meet clinical acceptability criteria. Additionally, most mean differences in *D*_*98*_, *D*_*95*_, *D*_*50*_, and *D*_*mean*_ between *DESIRE* and GT were below 1 Gy, with the mean differences in *D*_*2*_ being less than 1.6 Gy.

**TABLE 1:** Difference in dosimetric metrics between the proposed *DESIRE* model and ground truth (mean ± standard deviation) on testing cohort (*N* = 20).

		PTV70 (Gy)	PTV60 (Gy)	PTV55 (Gy)
*D*_*98*_	GT	69.79 ± 0.39	60.65 ± 0.70	54.11 ± 0.47
	PRED	68.79 ± 0.35	60.88 ± 0.98	54.03 ± 0.42
	*DIFF*	−1.00 ± 0.41	0.23 ± 0.76	−0.08 ± 0.64
	*p*	0.00	0.30	0.64
*D*_*95*_	GT	70.62 ± 0.69	61.68 ± 0.71	55.38 ± 0.46
	PRED	70.03 ± 0.08	62.13 ± 0.92	55.67 ± 0.53
	*DIFF*	−0.60 ± 0.71	0.45 ± 0.74	0.29 ± 0.71
	*p*	0.00	0.07	0.07
*D*_*50*_	GT	72.46 ± 0.53	69.24 ± 1.48	60.46 ± 1.99
	PRED	73.43 ± 0.82	69.88 ± 1.27	61.28 ± 1.82
	*DIFF*	0.97 ± 1.04	0.64 ± 0.76	0.82 ± 1.69
	*p*	0.00	0.05	0.08
*D*_*2*_	GT	73.90 ± 0.63	73.44 ± 0.59	73.33 ± 0.64
	PRED	75.45 ± 0.78	74.66 ± 0.68	74.55 ± 0.84
	*DIFF*	1.55 ± 0.87	1.22 ± 0.82	1.22 ± 0.88
	*p*	0.00	0.00	0.00
*D*_*mean*_	GT	72.27 ± 0.51	68.41 ± 0.82	62.45 ± 1.29
	PRED	73.19 ± 0.64	69.34 ± 0.96	63.18 ± 1.16
	*DIFF*	0.92 ± 0.82	0.93 ± 0.64	0.73 ± 0.67
	*p*	0.00	0.00	0.08

Abbreviations: *DIFF*, difference, calculated by subtracting GT from PRED; GT, ground truth; PRED, prediction of *DESIRE*.

Table 2 displays the *D*_1_ and *D*_*mean*_ for the OARs. Overall, there was no significant difference between the predictions of *DESIRE* and the GT in terms of both *D*_*1*_ and *D*_*mean*_. The mean difference in *D*_*mean*_ was around 1 Gy, and that in *D*_1_ was around 1.5 Gy.

**TABLE 2:** $D_{\text{mean}}$ and $D_{1}$ for OARs (mean ± standard deviation) on testing cohort (*N* = 20).

	*D*_*mean*_ (Gy)				*D*_*1*_ (Gy)			
	GT	PRED	*DIFF*	*p*	GT	PRED	*DIFF*	*p*
Brainstem	30.12 ± 6.12	30.94 ± 4.99	0.82 ± 3.44	0.88	50.76 ± 4.95	51.26 ± 4.82	0.50 ± 4.60	0.92
L eye	7.54 ± 3.42	8.22 ± 4.11	0.67 ± 2.17	0.84	21.13 ± 9.01	23.21 ± 11.34	2.09 ± 6.29	0.64
R eye	7.55 ± 3.19	8.25 ± 4.18	0.71 ± 2.96	0.92	21.61 ± 9.79	21.99 ± 10.49	0.38 ± 7.38	0.86
L len	4.98 ± 1.57	5.63 ± 2.53	0.65 ± 1.52	0.76	6.18 ± 2.34	7.72 ± 4.21	1.54 ± 2.72	0.39
R len	4.89 ± 1.33	5.99 ± 3.57	1.10 ± 3.12	0.80	5.95 ± 1.86	7.71 ± 4.91	1.76 ± 4.40	0.62
L middle ear	47.29 ± 7.92	48.35 ± 7.88	1.05 ± 2.98	0.54	65.16 ± 5.92	64.70 ± 6.44	−0.46 ± 2.32	0.74
R middle ear	42.15 ± 8.05	43.43 ± 7.94	1.27 ± 3.68	0.49	61.34 ± 6.32	60.76 ± 6.94	−0.58 ± 2.39	0.78
L optic nerve	25.17 ± 13.99	26.35 ± 13.97	1.17 ± 4.62	0.88	40.94 ± 18.62	43.92 ± 18.24	2.98 ± 5.66	0.69
R optic nerve	23.64 ± 12.55	25.39 ± 13.31	1.76 ± 4.22	0.68	39.16 ± 16.05	42.66 ± 16.88	3.50 ± 5.30	0.34
L parotid	36.07 ± 7.21	37.11 ± 6.55	1.05 ± 3.01	0.39	66.16 ± 5.46	65.63 ± 6.04	−0.53 ± 1.99	0.86
R parotid	34.92 ± 4.16	36.51 ± 4.14	1.59 ± 2.86	0.20	67.88 ± 5.64	67.50 ± 5.35	−0.38 ± 2.55	0.84
L temporal lobe	14.57 ± 4.27	14.48 ± 4.22	−0.09 ± 1.41	0.90	59.15 ± 5.67	60.42 ± 5.06	1.27 ± 2.84	0.39
R temporal lobe	14.14 ± 4.53	13.81 ± 4.45	−0.33 ± 0.92	0.78	57.03 ± 5.86	57.94 ± 6.54	0.90 ± 2.30	0.54
L TMJ	44.22 ± 8.18	44.17 ± 7.37	−0.05 ± 2.21	0.99	59.03 ± 8.28	58.45 ± 7.37	−0.58 ± 2.00	0.95
R TMJ	39.68 ± 8.02	40.31 ± 7.41	0.64 ± 2.80	0.62	53.83 ± 9.03	53.98 ± 8.40	0.14 ± 3.01	0.95
Larynx	39.85 ± 3.40	40.33 ± 2.88	0.48 ± 3.69	0.68	60.53 ± 5.21	60.30 ± 5.17	−0.23 ± 2.10	0.95
Optic chiasma	27.46 ± 13.60	29.67 ± 14.22	2.21 ± 4.67	0.52	37.73 ± 16.55	40.36 ± 16.17	2.63 ± 5.57	0.56
Oral cavity	37.93 ± 4.27	38.78 ± 3.73	0.85 ± 1.90	0.44	62.78 ± 6.59	62.55 ± 6.03	−0.23 ± 1.65	0.97
Spinal cord	27.34 ± 2.44	27.65 ± 2.68	0.31 ± 1.44	0.76	36.89 ± 1.01	37.78 ± 2.00	0.89 ± 2.20	0.11

Abbreviations: *DIFF*, difference, calculated by subtracting GT from PRED; GT, ground truth; PRED, prediction of *DESIRE*.

**FIGURE 4:**
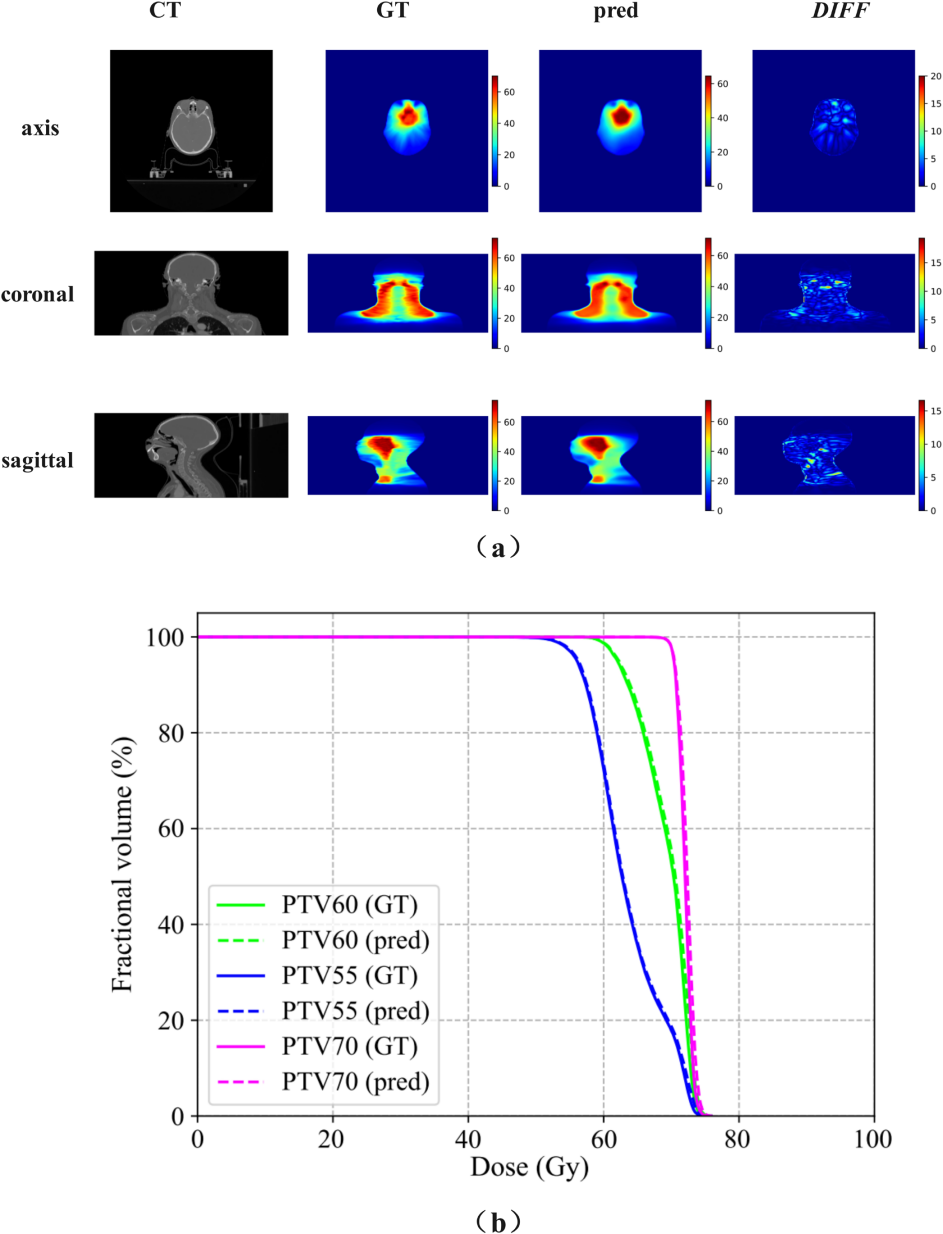
Comparison of ground truth (GT) and *DESIRE* predictions, (a) dose distribution, (b) dose–volume histogram (DVH).

To further visualize dose differences, the qualitative results of one case are shown in Figure 4, providing a more intuitive understanding of the model's predictive capabilities. The predicted dose distribution was relatively smooth, with minimal differences in individual pixels at the edges of some target areas, indicating no significant prediction errors. Figure 4b compares the GT DVHs of the PTVs and the DVHs predicted by the *DESIRE* for one patient in the test set. For all three PTVs, the dose curves predicted by *DESIRE* closely matched the real dose distribution curves, indicating satisfactory performance and clinical applicability.

### 3.2 Gamma Pass Rate for PTVs

**FIGURE 5:**
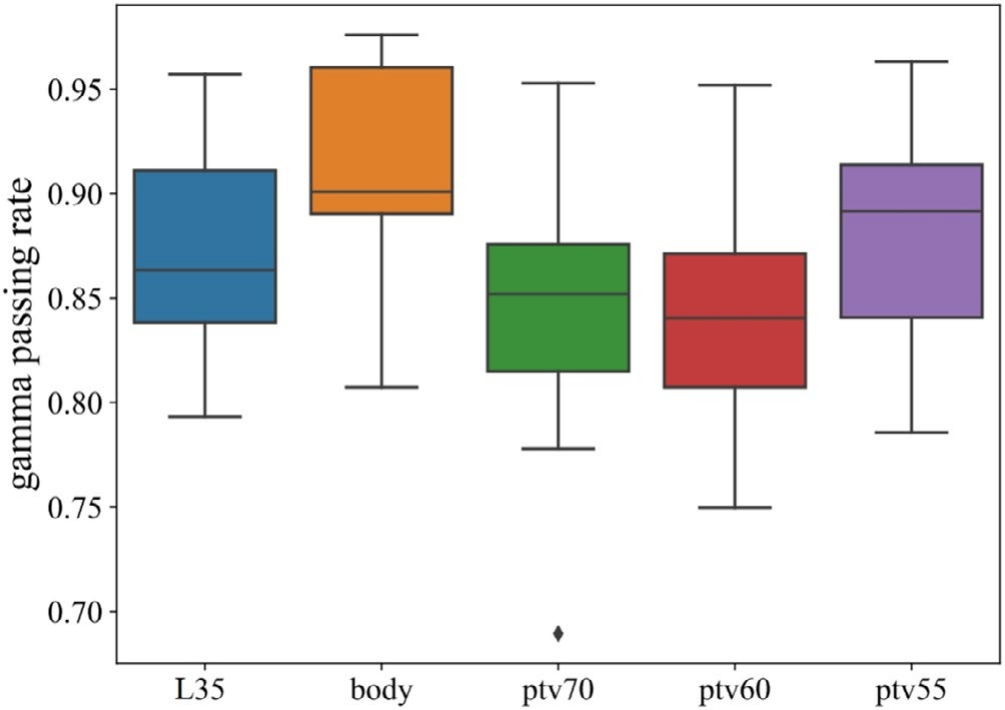
Gamma passing rate for L35, the body, PTV70, PTV60, and PTV55. L35: The region delineated by the 35‐Gy isodose line.

The PTVs in the 3D gamma analysis comprise the region delineated by the 35‐Gy isodose line (referred to as L35), the body, PTV70, PTV60, and PTV55. The box plot illustrating the gamma passing rate for PTVs is presented in Figure 5. The average passing rates were 87.4%, 91.3%, 84.7%, 84.1%, and 88.3%.

### 3.3 Comparison Between Different Models

To evaluate the performance of the proposed model, we compared it with several UNet‐based models, including 2D‐UNet, HD‐UNet, and 3D‐UNet. Table 3 presents the average absolute errors in dose prediction results for various PTVs and OARs of different models. The proposed *DESIRE* showed closer agreement with the ground truth for all PTVs, with a mean dose difference of 1.63 Gy in PTV60, 1.72 Gy in PTV55, and 1.43 Gy in PTV70, reducing the error by 0.26, 0.35, and 0.16, respectively, compared to the best results achieved by other models. In terms of OARs, although the differences for the Left/Right eyes and Left/Right lens were higher than those for HD‐UNet, no significant differences were observed (*p* > 0.05). Moreover, *DESIRE* showed closer agreement for all other OARs, although statistical significance was not achieved for all comparisons.

**TABLE 3:** Dose prediction error of different models (MAE) on testing cohort (mean ± standard deviation) (*N* = 20).

	*DESIRE*	2D‐Unet	HD‐Unet	3D‐Unet	*p*1	*p*2	*p*3
PTV60	**1.63 ± 0.29**	2.02 ± 0.83	2.38 ± 1.80	1.89 ± 0.69	**0.03**	**0.01**	0.21
PTV55	**1.72 ± 0.31**	2.07 ± 0.93	2.22 ± 1.75	2.14 ± 0.98	0.15	0.25	0.05
PTV70	**1.43 ± 0.33**	1.77 ± 0.89	2.69 ± 2.40	1.59 ± 0.73	0.11	**0.00**	0.86
Brain stem	**3.72 ± 2.12**	4.78 ± 2.46	4.46 ± 1.96	5.33 ± 2.26	0.09	0.10	**0.01**
Left eye	2.07 ± 1.43	2.79 ± 2.60	**1.75 ± 1.02**	2.24 ± 2.05	0.58	0.44	0.71
Right eye	2.31 ± 2.17	2.40 ± 1.56	**1.95 ± 1.80**	2.89 ± 3.11	0.52	0.62	0.80
Left lens	1.32 ± 1.07	2.24 ± 3.11	**0.98 ± 0.77**	1.10 ± 1.00	0.54	0.34	0.44
Right lens	1.56 ± 1.39	1.84 ± 1.98	**1.18 ± 1.69**	2.03 ± 3.32	0.84	0.16	0.27
Left middle ear	**3.11 ± 1.25**	3.74 ± 2.18	3.32 ± 1.77	4.00 ± 2.32	0.65	0.90	0.36
Right middle ear	**3.43 ± 1.48**	4.11 ± 2.28	4.32 ± 2.55	4.57 ± 2.15	0.48	0.41	0.10
Left optic nerve	**3.28 ± 2.12**	4.53 ± 2.85	3.76 ± 1.88	4.52 ± 2.43	0.11	0.32	0.08
Right optic nerve	**3.63 ± 1.84**	4.23 ± 2.61	3.67 ± 1.80	4.64 ± 2.99	0.78	0.99	0.52
Left parotid	**3.93 ± 0.98**	5.01 ± 1.58	4.01 ± 1.23	5.24 ± 1.95	0.06	0.88	**0.02**
Right parotid	**4.13 ± 1.56**	5.06 ± 1.77	4.55 ± 1.38	5.17 ± 1.90	0.10	0.30	0.09
Left temporal lobe	**1.70 ± 0.63**	2.10 ± 0.60	1.77 ± 0.56	2.06 ± 0.76	0.05	0.62	0.11
Right temporal lobe	**1.60 ± 0.68**	2.06 ± 0.72	1.91 ± 0.79	2.17 ± 1.03	**0.03**	0.20	0.09
Left TMJ	**2.79 ± 1.02**	3.09 ± 1.21	3.14 ± 1.06	4.46 ± 2.30	0.46	0.16	**0.00**
Right TMJ	**3.08 ± 1.34**	3.50 ± 1.50	3.61 ± 1.41	4.27 ± 2.28	0.41	0.25	0.06
Larynx	**3.38 ± 1.29**	4.35 ± 2.19	3.74 ± 1.95	4.53 ± 2.11	0.10	0.71	**0.04**
Optic chiasma	**4.31 ± 2.28**	5.53 ± 3.19	4.51 ± 2.82	7.23 ± 4.16	0.23	0.97	**0.02**
Oral cavity	**3.10 ± 1.37**	4.11 ± 1.33	4.00 ± 1.41	4.29 ± 1.62	**0.01**	**0.03**	**0.01**
Spinal cord	**2.17 ± 0.91**	2.52 ± 0.85	2.65 ± 1.03	2.77 ± 1.11	0.24	0.16	0.08

*Note:* The best results were highlighted in bold for each organ or region, with instances where *p* < 0.05 also emphasized in bold. Comparison details: *p*1, DESIRE vs. 2D‐Unet; *p*2, DESIRE vs. HD‐Unet; *p*3, DESIRE vs. 3D‐Unet.

Abbreviation: TMJ, temporomandibular joint.

### 3.4 Relevance of *DESIRE* in Plan Designing

The quality evaluation of the plans designed by two junior physicians is displayed in Table 4. The CI values for PTV70 of Planner 1 (fewer than 3 years of experience), Planner 2 (fewer than 5 years of experience), and GT (senior physician, over 10 years of experience) were 0.847, 0.845, and 0.827, respectively. All had a CI of 0.855 for PTV55. Planners 1 and 2 exhibited no significant differences in CI for both PTV70 and PTV55 when compared with the GT. Furthermore, in terms of HI for PTV70, Planner 2's performance surpassed that of the GT (0.049 vs. 0.057, *p* < 0.05). From the perspective of OARs, the performance of both planners was on par with the GT, except for Planner 1 in the brain stem (38.49 Gy vs. 37.56 Gy, *p* < 0.05) and larynx (41.74 Gy vs. 39.55 Gy, *p* < 0.05). For Planner 2, some OARs exceeded the GT values.

**TABLE 4:** The dosimetric metrics error between the different plans on testing cohort (mean ± standard deviation) (*N* = 20).

		GT	Planner 1 (< 3 years)	*p*1	Planner 2 (< 5 years)	*p*2
PTV70	*D*_*98*_	69.79 ± 0.39	69.66 ± 0.33	0.26	69.33 ± 0.28	**0.00**
	*D*_*95*_	70.62 ± 0.69	70.25 ± 0.30	**0.02**	70.06 ± 0.13	**0.00**
	*D*_*50*_	72.46 ± 0.53	72.07 ± 0.46	**0.02**	71.75 ± 0.37	**0.00**
	*D*_*2*_	73.90 ± 0.63	73.43 ± 0.64	**0.03**	72.96 ± 0.57	**0.00**
	*D*_*mean*_	72.27 ± 0.51	71.94 ± 0.45	**0.03**	71.61 ± 0.35	**0.00**
	CI	0.827 ± 0.037	0.847 ± 0.036	0.09	0.845 ± 0.029	0.14
	*HI*	0.057 ± 0.009	0.052 ± 0.008	0.11	0.049 ± 0.012	**0.04**
PTV60	*D*_*98*_	60.65 ± 0.70	60.26 ± 0.71	0.12	60.08 ± 0.64	**0.02**
	*D*_*95*_	61.68 ± 0.71	61.22 ± 0.72	0.05	61.15 ± 0.69	**0.02**
	*D*_*50*_	69.24 ± 1.48	68.85 ± 1.36	0.38	68.76 ± 1.34	0.22
	*D*_*2*_	73.44 ± 0.59	72.95 ± 0.63	**0.02**	72.63 ± 0.53	**0.00**
	*D*_*mean*_	68.41 ± 0.82	67.98 ± 0.76	0.12	67.89 ± 0.74	**0.02**
PTV55	*D*_*98*_	54.11 ± 0.47	54.23 ± 0.47	0.26	54.10 ± 0.34	1.00
	*D*_*95*_	55.38 ± 0.46	55.35 ± 0.35	0.88	55.27 ± 0.31	0.52
	*D*_*50*_	60.46 ± 1.99	59.73 ± 1.55	0.25	59.69 ± 1.96	0.12
	*D*_*2*_	73.33 ± 0.64	72.87 ± 0.56	**0.03**	72.50 ± 0.52	**0.00**
	*D*_*mean*_	62.45 ± 1.29	62.01 ± 1.06	0.22	61.83 ± 1.55	0.15
	CI	0.855 ± 0.025	0.855 ± 0.029	0.99	0.855 ± 0.023	0.82
Spinal_cord	*D*_*max*_	37.56 ± 1.06	38.49 ± 1.42	**0.03**	37.63 ± 1.46	0.92
Brain_stem	*D*_*max*_	52.51 ± 4.20	52.85 ± 2.68	0.96	51.89 ± 4.41	0.78
L_eye	*D*_*max*_	23.81 ± 10.01	24.66 ± 11.14	0.80	24.79 ± 11.54	0.97
R_eye	*D*_*max*_	22.61 ± 11.72	23.82 ± 10.37	0.78	24.18 ± 10.37	0.58
L_len	*D*_*max*_	5.69 ± 1.92	5.57 ± 1.66	0.85	5.74 ± 1.67	0.85
R_len	*D*_*max*_	5.67 ± 1.94	5.56 ± 1.68	0.92	5.83 ± 1.65	0.71
L_optic_nerve	*D*_*max*_	40.35 ± 17.30	40.40 ± 17.25	0.88	39.54 ± 17.55	0.59
R_optic_nerve	*D*_*max*_	40.21 ± 16.01	40.51 ± 16.23	0.95	39.60 ± 16.41	0.86
Optic_chiasma	*D*_*max*_	38.32 ± 15.91	38.03 ± 15.05	0.91	38.94 ± 15.46	0.97
L_tmj	*D*_*max*_	59.70 ± 8.20	58.90 ± 9.79	0.97	58.71 ± 8.35	0.60
R_tmj	*D*_*max*_	55.14 ± 8.93	54.43 ± 9.47	0.80	55.17 ± 8.91	0.90
L_middle_ear	*D*_*mean*_	46.01 ± 8.14	44.71 ± 6.24	0.52	45.08 ± 8.95	0.44
R_middle_ear	*D*_*mean*_	41.04 ± 8.09	41.34 ± 6.26	0.88	40.57 ± 8.09	0.75
Larynx	*D*_*mean*_	39.55 ± 3.46	41.74 ± 2.37	**0.03**	39.24 ± 3.32	0.56
Oral_cavity	*D*_*mean*_	37.00 ± 3.76	38.88 ± 3.77	0.12	36.57 ± 3.90	0.53
L_parotid	V_30_	48.48 ± 13.31	48.76 ± 13.05	0.78	47.16 ± 13.15	0.50
R_parotid	V_30_	46.55 ± 6.50	46.68 ± 6.56	0.89	44.90 ± 5.52	0.42

*Note:* Instances where *p* < 0.05 were emphasized in bold. Comparison details: p1, Planner 1 vs. GT; p2, Planner 2 vs. GT.

## 4 Discussion

Recent technological advances have caused radiotherapy to become highly complex, with software and hardware that are almost entirely dependent on human–computer interactions. Precise automated dose prediction holds the potential to substantially enhance both the efficiency and safety of clinical treatment planning. The results obtained from 3D dose prediction can be directly applied to the ongoing optimization of radiation therapy plans within the treatment planning system. In this study, we developed *DESIRE*. In routine clinical practice, variations in the planning process arise due to different physicians consulting with patients, introducing an element of uncertainty in radiation therapy planning results. Employing CNN‐based dose predictions to guide the plan optimization process can mitigate this uncertainty and concurrently enhance the efficiency of plan optimization.

In recent years, extensive research has applied deep learning to dose prediction tasks, with UNet and its variants predominating. Liu et al. [20] proposed a deep learning method for dose prediction based on the cascade mechanism and 3D UNet by taking advantage of data augmentations. They achieved MAE values of 2.50 Gy for a single C3D model without test‐time augmentation and a DVH score of 1.55. Using an attention‐gating mechanism and a 3D UNet for IMRT 3D dose distribution prediction in head and neck cancer, Osman et al. [11] also found that the average difference in predicting the *D*_*99*_ value for the targets was 2.50 ± 1.77 Gy. For the OARs, the average differences in predicting the *D*_*max*_ and *D*_*mean*_ values were 1.43 ± 1.01 Gy and 2.44 ± 1.73 Gy, respectively. The average value of the HI was 7.99 ± 1.45, and the CI was 0.63 ± 0.17 for the predicted plans. Nguyen et al. [10] proposed a 3D radiotherapy dose prediction method in head and neck cancer with a hierarchically densely connected UNet architecture and achieved an average prediction difference of maximum dose within 6.3% and mean dose within 5.1% of the prescription dose on test data across all OARs. Previous findings have demonstrated the potential and feasibility of UNet models. Despite their success, several aspects can be further improved for higher prediction accuracy. In this study, various strategies, such as progressive feature fusion and difficult region learning, were introduced. Our model underwent rigorous training and validation using a private dataset encompassing data from 131 patients with NPC. Comprehensive quantitative assessments have validated the model's clinical significance. Furthermore, we conducted comparative analyses with several state‐of‐the‐art UNet‐based methods, the *DESIRE* model demonstrated dose predictions that align closely with the ground truth generated by an experienced dosimetrist, further research is needed to assess whether *DESIRE*'s dose predictions result in optimal dose distributions and to evaluate its impact on plan quality and clinical outcomes.

Existing studies on dose prediction have mostly focused on improving the accuracy of dose prediction models while neglecting the evaluation of their true clinical applicability. In this study, we investigated how dose prediction can improve the quality of planning. Two junior physicians were invited to refer to the dose prediction results of *DESIRE* for planning design. The quality of the plans from these two physicians was comparable to that of the GT, designed by a senior physician with over 10 years of experience. The encouraging result demonstrates the clinical potential of using automatic planning, which not only enhances planning quality but also improves planning stability.

Patients with NPC typically experience long‐term survival. However, due to the involvement of a large number of OARs in NPC radiotherapy, effects on certain tissues, such as the brainstem, optic nerves, and larynx, largely influence patient quality of life. Thus, optimizing treatment plans to minimize OAR doses while adequately irradiating PTVs poses a challenge for treatment planners. In traditional optimization processes, planners manually set dose constraint values based on the spatial relationship between PTVs and OARs, taking into account the characteristics of different technologies. This iterative optimization strategy involves modifying parameters and applying various constraints based on optimization results to develop a plan that meets clinical requirements. However, this process is time‐consuming and heavily relies on planner expertise. Furthermore, achieving further reductions in healthy tissue doses necessitates continual experimentation with new dose constraints, increasing the workload.

Feasibility DVH [21] and KBP [22, 23] are two commonly used automated planning methods but with certain limitations. For example, feasibility DVH divides the normal tissue DVH into different regions (impossible, difficult, challenging, and probable); it does not provide specific dose distribution curves, and it does not take into account the interactions of different tissue doses in addition to the healthy tissue dose distribution, which is based on the premise that 100% of PTV covers the prescribed dose. In contrast, KBP‐based approaches do not adequately account for individual anatomical differences and specific clinical situations and rely on predefined models or templates based on population data. While these can provide valuable insight and general guidance for treatment planning, they may overlook nuances in a patient's anatomy or unique clinical factors that may affect treatment efficacy or toxicity outcomes. Consequently, there is a risk of creating suboptimal treatment regimens that do not meet the individual needs of each patient, which can lead to poor treatment outcomes.

Despite promising results, there are several limitations in this study. First, the framework predicts dose distributions only for VMAT plans and does not account for physician‐specific preferences, such as prioritizing OAR sparing over target homogeneity. Future research could address this by training preference‐aware models using data stratified by institutional protocols or physician planning styles, enabling tailored predictions for diverse clinical scenarios. Extending *DESIRE* to other technologies like IMRT or TOMO would require retraining with technology‐specific data to accommodate technical differences. Second, the model was validated on a homogeneous NPC cohort from a single institution. While *DESIRE* is adaptable to multi‐center data, variations in accelerator systems, TPS algorithms, and institutional constraints pose challenges. Future work will focus on expanding model validation across multiple institutions to assess its generalizability. Additionally, adapting *DESIRE* to other anatomical sites (e.g., lung, prostate) would require retraining with site‐specific datasets. Incorporating anatomical priors, such as tumor–OAR spatial relationships, would also be essential for improving model performance across different sites. Finally, while the model predicts 3D dose distributions, translating these predictions into actionable clinical plans remains unexplored. Integrating *DESIRE* with automated optimization engines could bridge this gap and enable end‐to‐end plan generation, making it clinically actionable. This will be a key direction for future research, aiming to enhance the model's clinical utility and streamline treatment planning.

## 5 Conclusions

Herein, we propose *DESIRE*, a *Dose prediction model with progrEssive feature fuSion and dIfficult Region lEarning*, for NPC treatment. This method has demonstrated its capability to provide highly accurate dose predictions. Unlike previous conventional DVH‐based prediction methods, our approach represents a highly promising advancement. The generated 3D dose map holds significant utility in enhancing the design of radiotherapy plans, ensuring their quality and consistency, and offering valuable guidance in the task of automatic treatment planning.

## Author Contributions

**Junming Jian:** conceptualization, funding acquisition, methodology, resources, software, validation, and writing – original draft. **Xingxing Yuan:** data curation, resources, supervision, and writing – review and editing. **Longfei Xu:** formal analysis, software, visualization, and writing – review and editing. **Changfei Gong:** data curation, investigation, and writing – review and editing. **Xiaochang Gong:** conceptualization, formal analysis, supervision, and writing – review and editing. **Yun Zhang:** conceptualization, funding acquisition, project administration, resources, supervision, and writing – review and editing.

## Ethics Statement

The studies involving human participants were reviewed and approved by the Ethics Committee of Jiangxi Cancer Hospital (Approval ID: 2022ky012). The study was conducted following the principles of the Declaration of Helsinki and in accordance with local statutory requirements.

## Conflicts of Interest

The authors declare no conflicts of interest.

## Data Availability

The data sets used during the current study are available from the corresponding author upon reasonable request.
